# Multiple roles of Activin/Nodal, bone morphogenetic protein, fibroblast growth factor and Wnt/β-catenin signalling in the anterior neural patterning of adherent human embryonic stem cell cultures

**DOI:** 10.1098/rsob.120167

**Published:** 2013-04

**Authors:** Giuseppe Lupo, Claire Novorol, Joseph R. Smith, Ludovic Vallier, Elena Miranda, Morgan Alexander, Stefano Biagioni, Roger A. Pedersen, William A. Harris

**Affiliations:** 1 Department of Physiology, Development and Neuroscience, University of Cambridge, Cambridge CB2 3DY, UK; 2 Department of Biology and Biotechnology ‘C. Darwin’, University of Rome ‘La Sapienza’, 00185 Rome, Italy; 3 Istituto Pasteur-Fondazione Cenci Bolognetti, University of Rome ‘La Sapienza’, 00185 Rome, Italy; 4 Department of Surgery and Anne McLaren Laboratory for Regenerative Medicine, Wellcome Trust and Medical Research Council Stem Cell Institute, University of Cambridge, Cambridge CB2 0SZ, UK

**Keywords:** human embryonic stem cells, neuroectoderm, anteroposterior patterning, forebrain, eye field

## Abstract

Several studies have successfully produced a variety of neural cell types from human embryonic stem cells (hESCs), but there has been limited systematic analysis of how different regional identities are established using well-defined differentiation conditions. We have used adherent, chemically defined cultures to analyse the roles of Activin/Nodal, bone morphogenetic protein (BMP), fibroblast growth factor (FGF) and Wnt/β-catenin signalling in neural induction, anteroposterior patterning and eye field specification in hESCs. We show that either BMP inhibition or activation of FGF signalling is required for effective neural induction, but these two pathways have distinct outcomes on rostrocaudal patterning. While BMP inhibition leads to specification of forebrain/midbrain positional identities, FGF-dependent neural induction is associated with strong posteriorization towards hindbrain/spinal cord fates. We also demonstrate that Wnt/β-catenin signalling is activated during neural induction and promotes acquisition of neural fates posterior to forebrain. Therefore, inhibition of this pathway is needed for efficient forebrain specification. Finally, we provide evidence that the levels of Activin/Nodal and BMP signalling have a marked influence on further forebrain patterning and that constitutive inhibition of these pathways represses expression of eye field genes. These results show that the key mechanisms controlling neural patterning in model vertebrate species are preserved in adherent, chemically defined hESC cultures and reveal new insights into the signals regulating eye field specification.

## 2. Introduction

Vertebrate neural development is initiated by the process known as neural induction that causes neuroectoderm formation in the dorsal region of the ectodermal germ layer [1]. During and/or following neural induction, the neuroectoderm is regionalized along the anteroposterior (AP) axis, leading to the specification of four major neural domains: forebrain, midbrain, hindbrain and spinal cord [2]. Each of these regions undergoes further patterning along both the AP and dorsoventral (DV) axes, forming the variety of cell populations present in the central nervous system (CNS). Diversification during neural patterning is particularly complex in the forebrain, where cells in nearby locations within the early neuroepithelium become differentially specified to give rise to structures such as the cerebral cortex, striatum, retina, thalamus and hypothalamus [3].

Studies performed in different model organisms have established that inhibition of bone morphogenetic protein (BMP) signalling, especially by extracellular antagonists such as Noggin and Chordin, is a necessary step in neural induction, whereas recent work has highlighted the importance of also repressing the Activin/Nodal pathway [4]. Although the role of fibroblast growth factors (FGFs) in neural induction is more controversial, evidence suggests that FGF signalling may act early to promote competence of the ectoderm towards neural induction and act later to reinforce intracellular inhibition of BMP signalling and/or repress transcription of BMP genes [5]. While BMP antagonism leads to induction of neuroectoderm with anterior positional identity [3], FGFs have been proposed to induce posterior neuroectoderm [6,7]. Moreover, exposure of rostral neuroectoderm to Wnts, FGFs and retinoic acid (RA) posteriorizes it to midbrain, hindbrain and spinal cord [3,8]. AP positional information is integrated with DV patterning mechanisms, mainly controlled by BMP and Wnt signalling (dorsally) and Hedgehog (Hh) signalling (ventrally; [9]). While significant headway has been made in elucidating the molecular mechanisms controlling neural patterning generally, regulation of forebrain regionalization remains poorly understood. For example, it is unclear how the segregation of the presumptive telencephalon and eye field, a major event in forebrain development [3], is achieved.

The establishment of human embryonic stem cell (hESC) culture has allowed analysis of these events *in vitro* in human tissue. Several groups have shown that key events in neural development, initially discovered within model organisms, can be replicated in this system. Examples include the neural-promoting actions of inhibitors of BMP and Activin/Nodal signalling [10–13] or of FGF signalling [13–15]. Furthermore, in the presence of low or absent BMP signalling, hESCs generate anterior neuroectoderm [16–18], which can be posteriorized by Wnt pathway activation or RA [16,19]. On this basis, several reports have described hESC specification to various neural fates, such as cerebral cortex [20,21], retina [22–24], midbrain dopaminergic neurons [19,25] or spinal cord motoneurons [26]. These studies have strikingly demonstrated that hESCs are amenable to the study of human CNS regionalization and production of specific neuronal types for subsequent applications. However, they have been mainly focused on cell-type production, rather than on the molecular mechanisms leading to cell fate specification, which have not been fully characterized. Moreover, while hESCs have been successfully steered to specific fates on the basis of pre-existing knowledge, they have been minimally exploited for gaining new insights into the regulation of neural patterning.

In this study, we have used defined culture conditions to systematically analyse the roles of the Activin/Nodal, BMP, FGF and Wnt/β-catenin pathways in neural induction, AP neural patterning and eye field specification in hESCs. We show how manipulation of these four pathways leads to specification of neuroectoderm with different AP identities ranging from forebrain to spinal cord. In addition, we show how such manipulation can be used to further influence forebrain patterning through regulation of eye field gene expression.

## 3. Material and methods

### 3.1. Human embryonic stem cell culture

H9 (WiCell Inc., Madison, WI, USA) hESCs (50–67 passages) were routinely expanded on irradiated mouse embryonic fibroblasts (MEFs) in knockout serum replacement (KSR) medium containing knockout Dulbecco's modified Eagle's medium (KO-DMEM, Invitrogen) supplemented with 20 per cent serum replacement (Invitrogen), 1 mM glutamine, 0.1 mM β-mercaptoethanol and 1 per cent non-essential amino acid stock, following published protocols [27]. MEF-free culture of hESCs in chemically defined medium (CDM) supplemented with Activin and FGF2 was performed as previously described [14,28]. The composition of CDM was 50 per cent Iscove's modified Dulbecco's medium (Invitrogen) plus 50 per cent F12 NUT-MIX (Invitrogen), supplemented with 7 µg ml^−1^ of insulin, 15 µg ml^−1^ of transferrin, 450 µM of monothioglycerol, 1 per cent chemically defined lipid concentrate (Invitrogen) and 5 mg ml^−1^ bovine serum albumin fraction V. To allow hESC adhesion in CDM, plates were precoated with 0.1 per cent porcine gelatin for 20 min followed by precoating with foetal bovine serum-containing medium (10% in KO-DMEM) for 24 h at 37°C, and then washed with phosphate buffered saline to eliminate any serum. For induction and patterning of neuroectoderm in adherent hESC cultures, the medium of hESCs grown in MEF-free conditions was replaced with CDM supplemented with specific molecular signals (small molecules and recombinant proteins), as described in §4 and the electronic supplementary material, table S1, and cells were cultured for 8–20 additional days, depending on the experiment. Small molecules were dissolved in dimethyl sulfoxide (DMSO), and equal volumes of DMSO were usually added to control cells. Karyotypic analyses and mycoplasma testing were performed on cells used for experiments without detecting abnormalities.

### 3.2. RT-PCR and real-time PCR

Total RNA was extracted from cultured cells using the Qiagen RNeasy Micro kit. RT-PCR was performed using the Qiagen OneStep RT-PCR kit. For real-time PCR, RNA was reverse-transcribed using the Qiagen QuantiTect reverse transcription kit and amplified on a Rotor-Gene Q (Qiagen), using Qiagen SYBR Green PCR kits. Primers for RT-PCR and real-time PCR were purchased from Qiagen. Relative gene expression levels in different samples were determined with the built-in comparative quantitation method [29] using *GAPDH* as a normalizer. Statistical analysis of experimental data was performed with Microsoft Excel software.

### 3.3. Immunofluorescence

Immunostaining on adherent hESC cultures was performed as previously described [11]. Images were captured with a Hamamatsu ORCA-ER camera mounted on a Nikon Eclipse 80i microscope, using Open*lab* software (Improvision). The following primary antibodies were used: mouse anti-NESTIN (1 : 200, R&D Systems); mouse anti-SOX2 (1 : 250, R&D Systems); rabbit anti-βIII-tubulin (1 : 200, Abcam); rabbit anti-PAX6 (1 : 300, Covance); mouse anti-CDX2 (1 : 250, Biogenex, donated by K. Niakin). Secondary antibodies used were Alexa Fluor 488 and 594 (1 : 500, Invitrogen).

## 4. Results

### 4.1. In adherent human embryonic stem cells, bone morphogenetic protein inhibition promotes anterior neuroectoderm specification, whereas fibroblast growth factor 2 causes induction of posterior neuroectoderm

Recent studies have described the induction of neuroectoderm in adherent hESC cultures by means of the inhibitor of Activin/Nodal signalling SB431542 (SB) and the BMP antagonist Noggin [12] or SB and FGF2 [14]. The neural inducing abilities of Noggin and FGF2, however, have not been directly compared. Thus, we performed side-by-side comparison of neuroectoderm specification with SB and Noggin or SB and FGF2, or with SB alone, in hESCs cultured in adherent, chemically defined conditions, as previously described [14,28].

**Figure 1.**
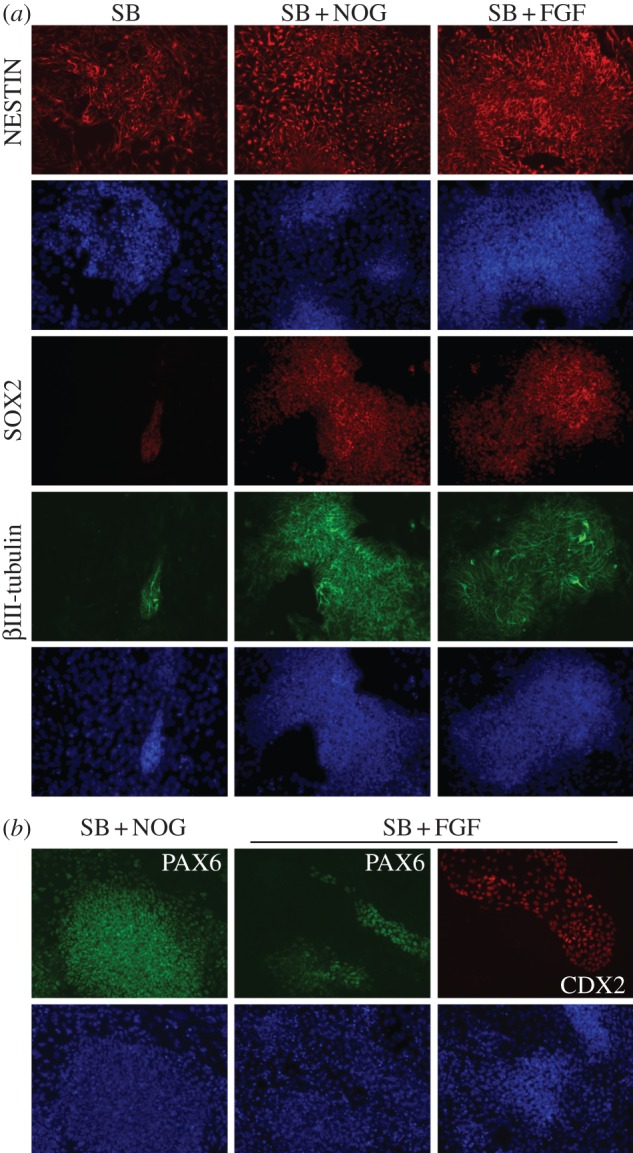
Treatments with SB + Noggin or SB + FGF2 promote neuroectoderm specification in adherent hESC cultures, but have opposite effects on the expression of PAX6 and CDX2. (*a*) Immunofluorescence analysis with NESTIN, SOX2 and βIII-tubulin antibodies in hESCs cultured for 14 days with SB, SB and Noggin (SB + NOG) or SB and FGF2 (SB + FGF), as indicated. Cells treated with SB alone broadly express NESTIN, but very few cells are positive for SOX2 compared with SB + NOG or SB + FGF treatments. SOX2-expressing cells are also positive for βIII-tubulin. DAPI blue staining shows cell nuclei. (*b*) Immunofluorescence analysis with PAX6 and CDX2 antibodies of hESCs cultured for 14 days with SB + NOG or SB + FGF. Compared with SB + NOG-treated cells, FGF2 treatments reduce the number of PAX6-positive cells and cause formation of CDX2-positive clusters.

**Figure 2.**
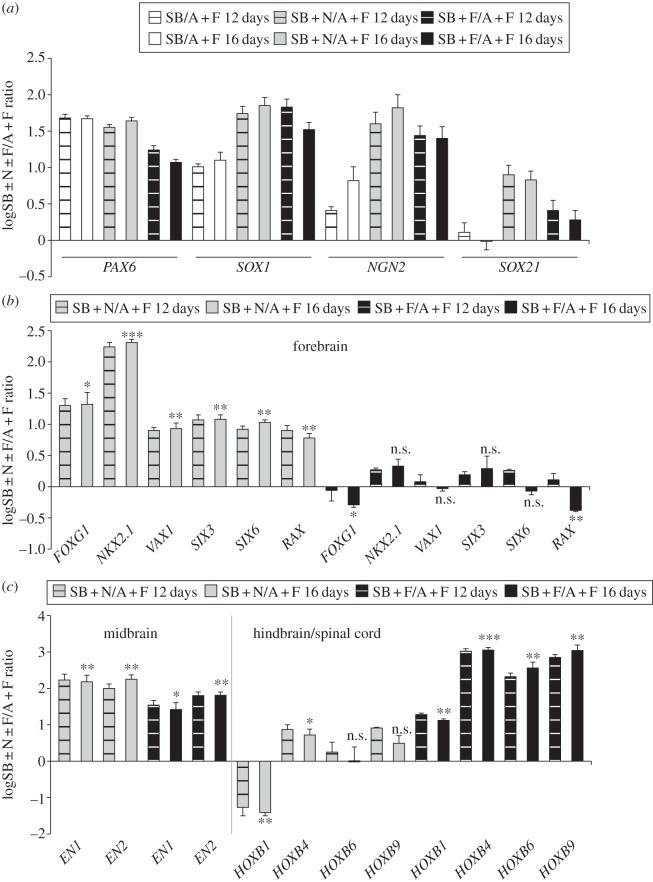
Treatment of adherent hESC cultures with SB + Noggin or SB + FGF2 induces neuroectoderm with different AP positional identities. (*a,b*) Real-time PCR quantification of gene expression in hESCs cultured for 12–16 days with SB, SB and Noggin (SB + N) or SB and FGF2 (SB + F), as indicated. Compared with SB treatments, SB + N or SB + F treatment enhances transcription of the neuroectoderm markers *SOX1*, *NGN2* and *SOX21*. However, cells treated with SB + N or SB + F show specific upregulation of forebrain or hindbrain/spinal cord genes, respectively. Upregulation of midbrain genes is detectable in both conditions. Results are shown as the mean of the log_10_-transformed ratio between SB, SB + N or SB + F and control conditions in three biological replicates. Undifferentiated hESCs cultured with Activin + FGF2 (A + F) were used as control. Error bars show the standard error of the mean. *p < 0.05; **p < 0.01; ***p < 0.001; n.s., non-significant (*p* ≥ 0.05) according to two-tailed Student's *t*-test performed between SB + N or SB + F and A + F conditions.

Immunofluorescence analysis in cells differentiated for 14 days with SB, SB + Noggin or SB + FGF2 using the neuroectoderm markers NESTIN, SOX2 and βIII-tubulin showed that the majority of cells cultured in any of these conditions were positive for the neural progenitor marker NESTIN (figure 1*a*). SB treatments, however, resulted in very few SOX2-positive cells compared with SB + Noggin or SB + FGF2 treatments (figure 1*a*). SOX2-expressing cells were also positive for the neural marker βIII-tubulin (figure 1*a*), confirming their neuroectodermal identity. These results were confirmed and extended by real-time PCR analysis of additional markers of neuroectoderm and non-neural cell fates. At 12–16 days of differentiation, cultures treated only with SB expressed similar or slightly lower levels of the neuroectodermal markers *NCAM* and *SOX3* to those treated with SB + Noggin or SB + FGF2 (see the electronic supplementary material, figure S1*a*), suggesting limited initiation of neural development even in the absence of Noggin or FGF2. These factors, however, caused clearly higher expression of the neural genes *SOX1*, *NGN2* and *SOX21* (figure 2*a*), indicating that Noggin or FGF2 are required for further progression of hESCs to neuroectoderm in these conditions. We found moderate upregulation of the neural crest marker *SOX10* and the non-neural markers *SOX7* and *CDX2* in SB-treated cells compared with undifferentiated cells, which was prevented by Noggin (see the electronic supplementary material, figure S1*a*,*b*). Similar to Noggin, FGF2 treatments restrained *SOX7* upregulation, whereas expression of pluripotency (*OCT4*) or mesendoderm (*SOX17*) markers was strongly downregulated in any of these conditions compared with undifferentiated cells (see the electronic supplementary material, figure S1*a* and data not shown). These results show that, in hESCs cultured in adherent, chemically defined conditions, downregulation of Activin/Nodal signalling initiates neural development to some extent, although in the presence of moderate transcription of non-neural markers, but inhibition of BMP signalling or upregulation of FGF signalling are clearly required to enhance the process of neural induction.

Treatments with SB + Noggin or SB + FGF2, although both superior to SB alone in terms of neural induction, had differential effects on AP patterning. Cultures treated with SB + Noggin showed larger areas of PAX6-positive cells compared with SB + FGF2 treatments, whereas CDX2-positive clusters were found in cells treated with SB + FGF2, but not SB + Noggin (figure 1*b*). These effects were confirmed by mRNA expression analysis (see figure 2*a*; electronic supplementary material, figure S1*a*,*b*). CDX2 expression has been associated with trophoectoderm and/or mesoderm differentiation in hESCs [30,31]. Thus, we analysed the expression of the trophoectoderm markers *EOMES* and *HAND1* and of the mesoderm markers *T*, *FLK1*, *LMO2* and *TBX6* in cultures treated with SB + FGF2 and found that *EOMES*, *HAND1*, *T* and *FLK1* were all downregulated in SB + FGF2-treated cells compared with undifferentiated cells, whereas *LMO2* and *TBX6* were weakly upregulated (see the electronic supplementary material, figure S2*a*,*b*). As Pax6 expression is more abundant in the anterior regions of the embryonic neuroectoderm [32], whereas Cdx2 is involved in posterior neural development [33], a possible explanation for the opposite regulation of PAX6 and CDX2 expression in cultures treated with SB + Noggin and SB + FGF2 might be that Noggin and FGF2 affect positional information differentially.

We therefore investigated the positional identity of neuroectoderm generated by SB + Noggin or SB + FGF2 treatments. Real-time PCR analyses of markers of AP neural patterning showed clear differences between gene expression profiles of cultures treated with SB + Noggin or SB + FGF2 for 12–16 days. Cells treated with SB + FGF2 showed no significant upregulation of the forebrain markers *FOXG1*, *NKX2.1*, *VAX1*, *SIX3*, *SIX6* and *RAX* compared with undifferentiated cells, whereas the hindbrain/spinal cord markers *HOXB1*, *HOXB4*, *HOXB6* and *HOXB9* were all robustly upregulated (figure 2*b*,*c*). By contrast, SB + Noggin treatments significantly upregulated forebrain markers, but not hindbrain/spinal cord markers, with the exception of a slight activation of *HOXB4* (figure 2*a,b*). The midbrain markers *EN1* and *EN2* were significantly upregulated in both conditions (figure 2*c*). In conclusion, SB + FGF2 treatments resulted in the specification of neuroectoderm with intermediate and posterior (midbrain/hindbrain/spinal cord) positional identities, whereas SB + Noggin-treated cells exhibited more anterior (forebrain/midbrain) gene expression profiles. These results are consistent with recent studies showing that adherent hESCs acquire anterior neuroectodermal fates in the presence of BMP and Activin/Nodal inhibitors [17,18].

### 4.2. Neuroectoderm posteriorization in adherent human embryonic stem cell cultures is dependent on Wnt/β-catenin and fibroblast growth factor signalling

**Figure 3.**
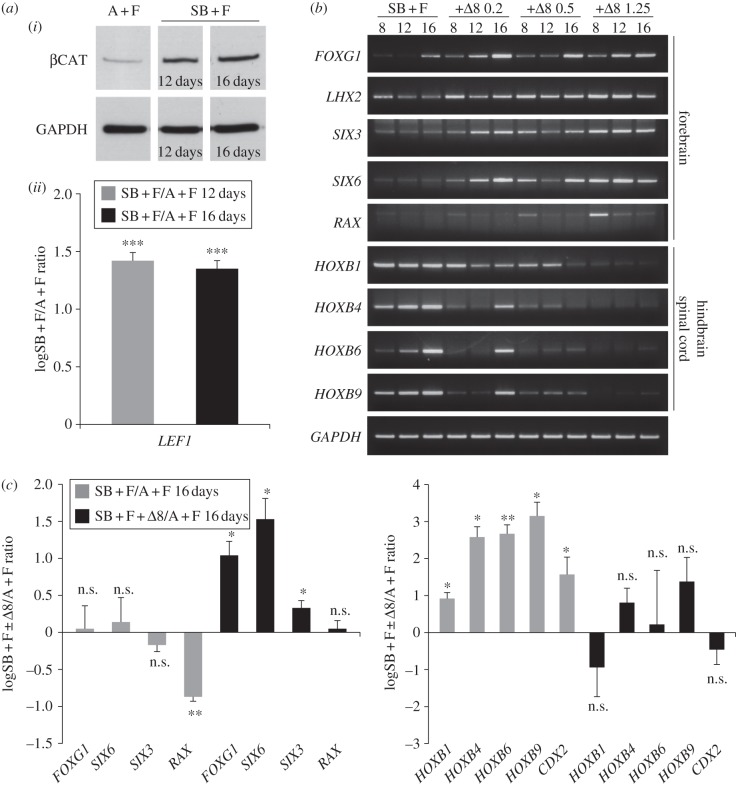
Endogenous Wnt signalling is upregulated in SB + FGF2-treated cultures and causes neuroectoderm posteriorization. (*a*) (i) Western blot analysis of β-catenin (βCAT) expression in cytosolic extracts from hESCs cultured with A + F, or hESCs differentiated for 12 or 16 days with SB + F. GAPDH was used as a loading control. (ii) Real-time PCR quantification of *LEF1* expression in hESCs treated for 12–16 days with SB + F. Results are shown as the mean of the log_10_-transformed ratio between SB + F and A + F conditions in six biological replicates. Both assays show Wnt pathway activation during hESC differentiation with SB + F. (*b*) RT-PCR analysis of gene expression in hESCs treated for 8–16 days with SB + F or with SB + F and increasing doses (0.2–1.25 µg ml^−1^) of Fzd8*Δ* (*Δ*8), showing that treatments with Wnt antagonists can reverse the expression profiles of AP neural markers compared with SB + F-treated cells. *GAPDH* was used as a loading control. (*c*) Real-time PCR quantification of gene expression in hESCs treated for 16 days with SB + F or with SB + F plus 0.5 µg ml^−1^ *Δ*8, showing that Wnt antagonism upregulates forebrain genes and reduces hindbrain/spinal cord gene expression in SB + F-treated cells. Results are shown as the mean of the log_10_-transformed ratio between SB + F or SB + F + *Δ*8 and A + F conditions in three to six biological replicates.

During embryonic development, posterior neural patterning is mainly controlled by the Wnt/β-catenin, FGF and RA signalling pathways [8,34]. As our culture conditions did not contain retinoids, we focused on the roles of Wnt/β-catenin and FGF signalling in AP patterning of hESC-derived neuroectoderm. Western blot analysis of the levels of cytosolic β-catenin showed that they were low in undifferentiated hESCs, but strongly increased following 12–16 days of differentiation with SB + FGF2 (figure 3*a*). *LEF1* is a direct target of Wnt/β-catenin signalling that can be used as a readout of β-catenin activity [19]. Its levels were also robustly increased in SB + FGF2-treated cells (figure 3*a*). To assess whether Wnt/β-catenin pathway activation is instrumental in FGF2-induced posteriorization, we used a soluble form of the extracellular domain of Frizzled8 (Fzd8*Δ*), which sequesters Wnt ligands and prevents activation of endogenous receptors [35]. When Fzd8*Δ* was added to differentiating hESCs together with SB + FGF2, it rescued forebrain gene expression and counteracted upregulation of hindbrain/spinal cord genes with dose-dependent effects (figure 3*b*). Real-time PCR analysis after 16 days of differentiation with intermediate doses of Fzd8*Δ* (500 ng ml^−1^) confirmed a shift in the gene expression profiles of neuroectoderm generated with SB + FGF2 from posterior to anterior (figure 3*c*). Similar results were obtained using Dkk1 as a Wnt antagonist (data not shown). Conversely, treatments with Wnt3a together with SB + FGF2 accelerated neuroectoderm posteriorization (see the electronic supplementary material, figure S3).

β-Catenin-independent Wnt signalling is also implicated in the regulation of neural plate patterning by opposing the β-catenin-dependent pathway [36,37]. Wnt5a, for example, can activate β-catenin-independent signalling [38]. Different from Wnt3a, treatments with Wnt5a could partially reverse the posterior identity of SB + FGF2-treated cells (see the electronic supplementary material, figure S3), indicating that the balance between β-catenin-dependent and β-catenin-independent Wnt signalling can influence AP patterning of hESC-derived neuroectoderm.

**Figure 4.**
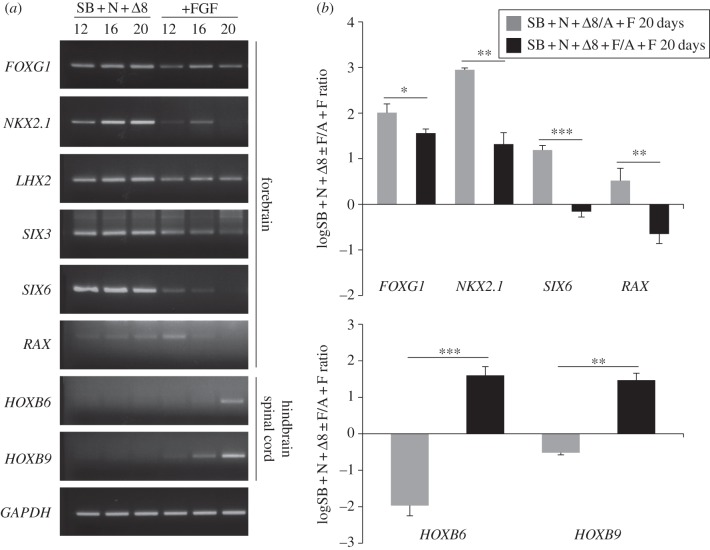
Exogenous FGF2 has a posteriorizing influence on neuroectoderm derived from hESC adherent cultures. (*a*) RT-PCR analysis of gene expression in hESCs treated for 12–20 days with SB, Noggin and Fzd8*Δ* (SB + N + *Δ*8), with or without FGF2, indicating that exogenous FGF2 can exert a partial posteriorizing effect on cells treated with Wnt antagonists. (*b*) Real-time PCR quantification of gene expression in hESCs treated for 20 days with SB + N + *Δ*8, with or without FGF2 (F), showing improved expression of forebrain genes and downregulation of posterior neural genes in the absence of exogenous FGF2 compared with FGF2-treated cells. Results are shown as the mean of the log_10_-transformed ratio between SB + N + *Δ*8 or SB + N + *Δ*8 + F and A + F conditions in four biological replicates. Two-tailed Student's *t*-test was performed between SB + N + *Δ*8 and SB + N + *Δ*8 + F conditions.

FGF signalling is involved in the specification of posterior neural fates in various animal model systems [6,8,39]. We therefore decided to examine the contribution of exogenous FGF2 to AP patterning of hESC-derived neuroectoderm. To this aim, we first differentiated adherent hESCs in the presence of SB, Fzd8*Δ* and increasing concentrations of FGF2, and found dose-dependent upregulation of hindbrain/spinal cord gene expression when FGF2 doses were elevated (see the electronic supplementary material, figure S4). Thus, exogenous FGF2 is able to promote posterior gene expression even when Wnt/β-catenin signalling is inhibited. These results prompted us to assess whether, conversely, completely avoiding exogenous FGF2 could improve forebrain specification in the presence of Wnt antagonists. We then used Noggin in place of FGF2 for neural induction and treated adherent hESCs with SB, Noggin and Fzd8*Δ*, in the absence or in the presence of low doses of FGF2 (12 ng ml^−1^). Analyses of markers of AP neural patterning after 12–20 days of differentiation showed clear differences between cells cultured with or without FGF2. Several forebrain markers, and especially *NKX2.1* and *SIX6*, were more robustly expressed in cells not exposed to FGF2 (figure 4*a,b*), whereas residual expression of the caudal markers *HOXB6* and *HOXB9* was abrogated (figure 4*a,b*). Therefore, in adherent hESCs differentiated into neuroectoderm with SB + FGF2, both upregulation of Wnt/β-catenin signalling and exogenous FGF2 impair forebrain specification and promote posterior neural fates.

**Figure 5.**
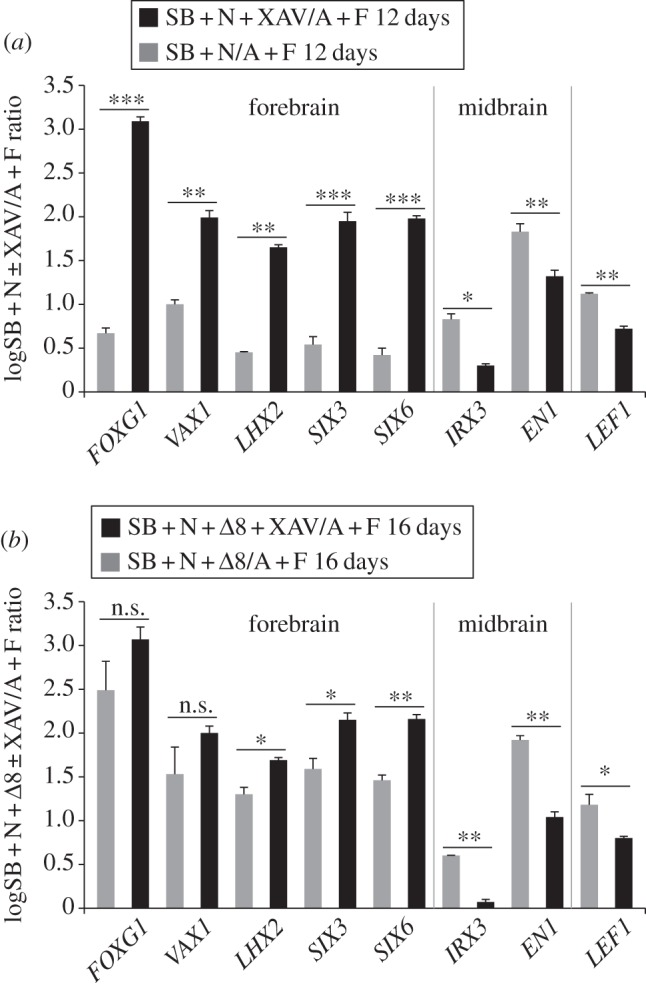
β-Catenin-dependent signalling restrains forebrain and promotes midbrain gene expression in anterior neuroectoderm derived from adherent hESC cultures. (*a,b*) Real-time PCR quantification of gene expression in hESCs treated for 12 days with SB + N, with or without XAV (*a*), or in hESCs treated for 16 days with SB + N + *Δ*8, with or without XAV (*b*). In both cases, XAV treatments resulted in the improved expression of forebrain genes and downregulation of midbrain genes and *LEF1*. Results are shown as the mean of the log_10_-transformed ratio between cells differentiated with or without XAV and undifferentiated hESCs in three biological replicates, with the exception of SB + N + *Δ*8 condition for which two replicates were used. Two-tailed Student's *t*-test was performed between cells differentiated with or without XAV.

As described earlier, neuroectoderm induced by treatment of adherent hESCs with SB + Noggin, while mostly anterior, shows a mild degree of posteriorization, as reflected by the expression of midbrain markers along with forebrain-specific genes (figure 2*c*). *LEF1* was upregulated in hESCs differentiated with SB + Noggin compared with undifferentiated cells (figure 5*a*). Upregulation of the midbrain markers *IRX3* and *EN1*, however, was still detectable after exposure to Fzd*Δ*8 (figure 5*b*). This suggests that midbrain specification may be mediated by activation of β-catenin signalling independently, at least in part, of Wnt signals. To test this idea, we took advantage of the small molecule XAV939 (XAV) that promotes β-catenin degradation [40]. When SB + Noggin-treated cultures were also exposed to XAV, *LEF1* was significantly downregulated (figure 5*a*). Moreover, XAV treatment enhanced forebrain-specific gene expression and decreased activation of midbrain markers (figure 5*a*), indicating that midbrain specification in SB + Noggin-generated neuroectoderm is β-catenin-dependent. XAV treatments exerted similar effects even in cultures treated with SB + Noggin + Fzd*Δ*8 (figure 5*b*), suggesting that, in adherent hESCs differentiating to neuroectoderm, Wnt-independent activation of β-catenin signalling may contribute to restrain forebrain specification and promote posterior neural fates.

### 4.3. Endogenous Hedgehog signalling is active in adherent human embryonic stem cell cultures and promotes neuroectoderm ventralization

While this work was mainly focused on the molecular mechanisms controlling AP neural patterning, we also observed that in hESCs differentiated into anterior neuroectoderm the ventral markers *NKX2.1* and *VAX1* were upregulated, whereas the dorsal marker *EMX1* was not detectable (see the electronic supplementary material, figure S5 and data not shown), indicating partial ventralization of the induced forebrain. In support of this interpretation, we found that Hh signalling was active in adherent hESCs differentiating to anterior neuroectoderm and that this promoted *NKX2.1* expression (see the electronic supplementary material, figure S5). Inhibition of the Hh pathway, while preventing *NKX2.1* expression, neither repressed *VAX1* nor activated *EMX1* expression, indicating that abrogation of Hh signalling is not sufficient for dorsalization of forebrain fates (see the electronic supplementary material, figure S5 and data not shown). While future work will be needed to study the molecular mechanisms of DV patterning in this system, these preliminary data provide a proof of principle that hESC-derived neuroectoderm in adherent, defined conditions is competent for regionalization along the DV axis.

### 4.4. Constitutive inhibition of Activin/Nodal and bone morphogenetic protein signalling hinders eye field specification in adherent human embryonic stem cells

In the experiments described earlier, we note that, even in the most favourable conditions for forebrain specification, eye field markers, such as *RAX* and *VSX2* [41], were rarely upregulated, suggesting that constitutive inhibition of Activin/Nodal and/or BMP signalling was not permissive for eye field specification or that other factors were missing. To test the former hypothesis, we performed a systematic analysis of the effects of SB and Noggin treatments on eye field specification. We first differentiated adherent hESC cultures in the presence of Fzd8*Δ* (to facilitate anterior neural specification) and different combinations of SB and Noggin for 12–16 days, followed by expression analysis of markers of general neuroectoderm, brain-specific anterior neuroectoderm (telencephalon/diencephalon) and eye field. *LEFTY2* and *ID1* are direct target genes of Activin/Nodal and BMP signalling [42,43], respectively, which we used to show that these pathways were effectively inhibited when SB or Noggin were applied (see the electronic supplementary material, figures S7*a* and S8*a*).

**Figure 6.**
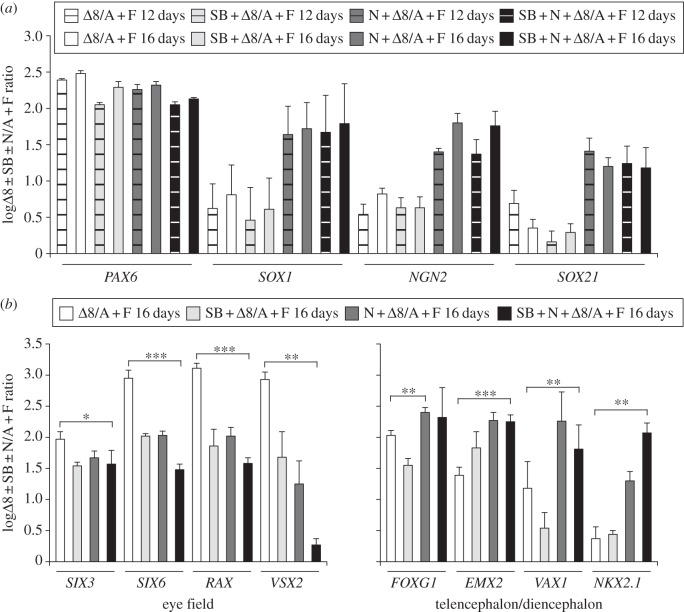
Activin/Nodal and BMP antagonists hinder eye field specification in anterior neuroectoderm derived from adherent hESC cultures. (*a,b*) Real-time PCR quantification of gene expression in hESCs treated for 12–16 days with *Δ*8, with or without SB and/or Noggin, as indicated. Results are shown as the mean of the log_10_-transformed ratio between differentiated and undifferentiated hESCs in four biological replicates. (*a*) Noggin, but not SB, treatments enhance transcription of the neuroectoderm markers *SOX1*, *NGN2* and *SOX21*. (*b*) Expression of eye field genes is repressed by SB and Noggin treatments, which instead promote expression of telencephalic/diencephalic genes. Two-tailed Student's *t*-test was performed between cells differentiated with or without SB and Noggin.

In the absence of both SB and Noggin, hESCs differentiating in adherent, chemically defined conditions initiated neural development to a limited extent, upregulating *NCAM* and *SOX3* at similar or slightly lower levels to those detected in cells treated with SB and Noggin (see the electronic supplementary material, figure S6*a*,*b*). Analysis of several other neural and non-neural markers showed that Noggin caused much more robust neural induction, which was not further improved by SB treatment (see figure 6*a* and electronic supplementary material, figure S6*a,b*). SB and Noggin specifically interfered with activation of genes expressed in the developing eye field, such as *PAX6*, *SIX3*, *SIX6*, *RAX*, *VSX2* and *LHX2* [41], as shown by the significantly higher expression levels of these genes in cells differentiated without these factors (figure 6*a*,*b* and data not shown). The inhibitory action of SB and Noggin on eye field genes was additive, because cells treated only with SB or Noggin had intermediate expression levels compared with cells treated with both, or with neither, of these factors (figure 6*b*). Different from eye field genes, expression of the telencephalic/diencephalic markers *FOXG1*, *EMX2*, *VAX1* and *NKX2.1* was significantly enhanced by treatments with SB and/or Noggin (figure 6*b*). These results suggest that treatment of adherent hESCs with SB and Noggin allows the specification of anterior neuroectoderm-showing expression of telencephalic-/diencephalic-specific genes, but represses specification of eye field fates.

By inhibiting the Activin/Nodal pathway during different phases of eye field specification, we began to probe when this repression occurs. Some cultures were differentiated with Noggin and Fzd8*Δ* for 12 days and treated with SB from the start of differentiation until 4, 6 or 8 days, after which SB was washed out and replaced with low doses of Activin (5 ng ml^−1^) until day 12 to restore Activin/Nodal signalling. Other cultures were instead exposed to SB only from day 4, day 6 or day 8 until day 12. Analysis of eye field genes showed that *RAX* and *LHX2* were mainly repressed by SB during the first 4 days of differentiation (see the electronic supplementary material, figure S7*b*). Instead, *SIX6* and *VSX2* were partially repressed when cells were treated with SB either from day 0 to day 4, day 6 or day 8 of differentiation or from day 4, day 6 or day 8 until day 12 of differentiation, indicating prolonged sensitivity to Activin/Nodal inhibition (see the electronic supplementary material, figure S7*c*). *LEFTY2* expression decreased following Activin and FGF2 removal and differentiation in Noggin + Fzd8*Δ*, but it was much more strongly downregulated in cells treated with SB (see the electronic supplementary material, figure S7*a*), indicating that low levels of Activin/Nodal signalling persist in cultures differentiated without SB, even after removal of exogenous Activin. When SB was applied only during the first 4–8 days of culture and replaced with Activin, *LEFTY2* expression levels at day 12 were similar to those in cultures never exposed to SB (see the electronic supplementary material, figure S7A), indicating that these low doses of Activin restored comparable levels of Activin/Nodal signalling with those present in Noggin + Fzd8*Δ*-treated cells.

A similar analysis was carried out for the BMP pathway using Noggin as an inhibitor and low doses of BMP4 (5 ng ml^−1^) to restore BMP signalling at specific time points. Analysis of eye field genes showed that *SIX6* and *VSX2* were mainly repressed by Noggin after the first 6–8 days of differentiation (see the electronic supplementary material, figure S8B). Expression of the telencephalic/diencephalic genes *NKX2.1* and *VAX1* was also regulated by Noggin during specific time windows. Noggin mainly promoted *NKX2.1* expression during the first 4 days of differentiation and *VAX1* expression after the first 6–8 days of differentiation (see the electronic supplementary material, figure S8*c*). The neuroectoderm markers *SOX1* and *SOX2* were activated by time-restricted Noggin treatments at intermediate levels between those detected in constitutive treatments and in the absence of Noggin (see the electronic supplementary material, figure S8*b*,*c*). *ID1* expression increased during differentiation in SB + Fzd8*Δ*, but it was downregulated in cells treated with Noggin (see the electronic supplementary material, figure S8*a*), indicating endogenous activation of BMP signalling in cultures differentiated without Noggin. When Noggin was applied only during the first 4–8 days of culture and replaced with BMP4, *ID1* expression levels at day 12 were similar to those of cultures never exposed to Noggin (see the electronic supplementary material, figure S8*a*), indicating that these low doses of BMP4 restored comparable levels of BMP signalling with those present in SB + Fzd8*Δ*-treated cells.

In conclusion, these assays showed that Activin/Nodal inhibition starts to interfere with eye field specification during the first 4 days of differentiation, whereas BMP inhibition hampers eye field gene expression mostly after the first 6–8 days of differentiation.

## 5. Discussion

**Figure 7.**
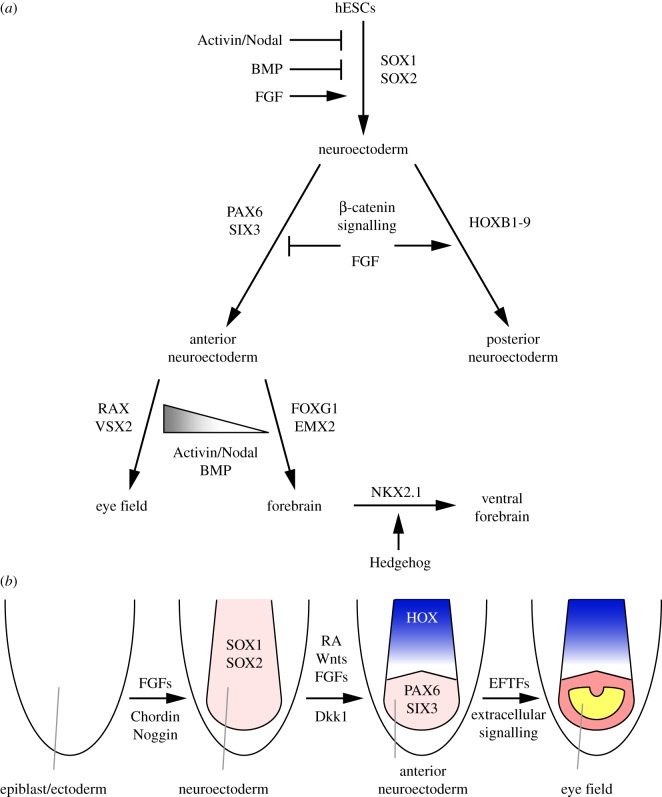
Proposed models of the signalling pathways controlling neural induction, AP neural patterning and further forebrain regionalization in adherent, chemically defined hESC cultures (*a*) or in a vertebrate embryo (*b*). Neural induction is promoted by BMP antagonists (e.g. Chordin, Noggin) and FGFs and is inhibited by high levels of Activin/Nodal signalling. AP patterning is controlled by posteriorizing factors (RA, Wnts and FGFs) and their antagonists (e.g. Dkk1). Eye field specification is marked by upregulation of eye field-specific transcription factors (EFTFs) and, at least in hESCs, involves higher levels of Activin/Nodal and BMP signalling compared with forebrain specification (grey gradient in (*a*)). Key transcription factors regulated during these events are also indicated.

In this study, we performed a systematic analysis of the roles of Activin/Nodal, BMP, FGF and Wnt/β-catenin signalling in neural induction and patterning of hESCs cultured in adherent, chemically defined conditions. As schematized in figure 7, we show that inhibition of BMP signalling or activation of FGF signalling are required for effective neural induction in this system but have strikingly different outcomes in terms of the AP identity of the induced neuroectoderm. We demonstrate that specification of positional fates posterior to forebrain is dependent on Wnt/β-catenin and FGF signalling and that inhibition or minimization of these pathways is needed for efficient forebrain specification in our culture conditions. We show that further forebrain patterning is influenced by the levels of Activin/Nodal and BMP signalling and that constitutive inhibition of these pathways suppresses eye field specification.

Studies in model organisms have shown that BMP inhibition is a critical step in neural induction [4]. This notion was initially suggested by the ability of BMP antagonists to neuralize *Xenopus* ectodermal explants [44], but has then been validated by work showing that neural induction is enhanced in embryos with defective BMP signalling [45–47]. BMP inhibition, however, is not sufficient to neuralize *Xenopus* ventral ectoderm (prospective epidermis) *in vivo*, which requires simultaneous inhibition of Activin/Nodal signalling [48]. Correspondingly, mice *Nodal* mutants display premature neural induction [49].

Our work confirms recent studies demonstrating the necessity of inhibiting BMP signalling for effective neuroectoderm specification in hESCs [10,12,13,18]. There are, however, also noteworthy differences. Previous work using adherent hESC cultures suggests that downregulation of Activin/Nodal signalling without BMP antagonism in hESCs predominantly causes trophoblast differentiation [30], whereas inhibition of both Activin/Nodal and BMP pathways is needed for efficient neuralization of hESCs [12,18]. We show instead that adherent hESC cultures differentiated in the absence of BMP antagonists were able to initiate neural development to some extent, as they contained large numbers of NESTIN-positive cells and upregulated other early neural markers, such as *NCAM* and *SOX3*, although in the presence of moderate upregulation of non-neural markers. Noggin inhibition was required to erase upregulation of non-neuroectodermal genes and promoted a much more robust programme of neural specification including upregulation of *SOX1*, *NGN2*, *SOX21* and generation of SOX2/βIII-tubulin-positive cells. Surprisingly, however, the Activin/Nodal inhibitor SB had negligible effects on Noggin-treated cultures, suggesting that hESCs differentiated in chemically defined conditions are in a similar situation to *Xenopus* ectodermal explants, where the levels of Activin/Nodal signalling are sufficiently low to allow neuroectoderm specification when BMP signalling is inhibited. Differences with previous reports could be due to inclusion in studies by others of additional reagents, such as feeder-conditioned medium, matrigel or serum replacements, which may result in higher levels of endogenous Activin/Nodal and/or BMP signalling than those present in our cultures. Given the ability of SB treatments to influence forebrain patterning in embryoid bodies [50] or in adherent cultures (this study), achieving neural induction in the absence of Activin/Nodal inhibitors may be crucial for protocols aimed at driving hESCs towards specific forebrain fates. Activin/Nodal inhibitors, however, remain useful in hESCs neuralized with FGF2, because pluripotency markers were downregulated more slowly in cultures treated with FGF2 without SB (data not shown), in agreement with Activin/Nodal and FGF cooperation in pluripotency [28]. The requirements of adherent hESCs for neural development seem partially different from those of embryoid body cultures, where neural induction can happen without exogenous BMP antagonists [13] and is enhanced by Activin/Nodal inhibitors [11], indicating that production of Activin/Nodal and BMP ligands or antagonists is different in embryoid bodies as compared with adherent cultures.

Several studies have investigated the role of FGF signalling in neural induction, but conflicting findings have been reported depending on the model system and experimental conditions used. Work supporting the relevance of FGF signalling in neural induction has suggested that FGFs may promote competence of the ectoderm towards neuralization and/or reinforce neural specification by repressing BMP ligand transcription or intracellular signal transduction [1,5]. It has also been proposed that FGFs may independently induce posterior neuroectoderm [7,6]. While the role of FGF signalling in neural induction remains not fully understood, including the specific ligands and receptors involved, there is a more general consensus on its role, together with Wnt signals and RA, as a posteriorizing factor of neuroectoderm induced by BMP antagonists [2,3].

Even in the field of hESCs, the role of FGF signalling in neural induction is controversial, with recent studies suggesting that it promotes [13–15] or inhibits [51] neural specification. Supporting a role for FGF in hESC neuralization, we show that treatment of adherent hESCs with FGF2 along with SB strongly enhanced neuroectoderm specification and elicited effects comparable with those achieved with Noggin. While it was previously shown that the protocols used in this study result in nearly homogeneous neuralization of hESCS after one week of differentiation [14], at the later time points used for this study (12–16 days), we found that both SB + Noggin-treated cultures and SB + FGF2-treated cultures contained substantial numbers of SOX2-negative cells (data not shown), even though these cultures showed weak, if any, upregulation of the various non-neural markers that we tested. We speculate that during the second week of differentiation a significant proportion of cells do not maintain their neural fate in spite of the presence of FGF2 or Noggin, without acquiring mesendodermal or extraembryonic fates. The reasons for the emergence of heterogeneous cell populations from initially homogeneous cultures could be explained by the presence of Notch-dependent lateral inhibition mechanisms [52], by fluctuations in exogenous growth factor signalling owing to their instability in the culture medium [53], and/or by unequal exposure to exogenous factors as colonies grow bigger during differentiation.

The most remarkable differences between Noggin and FGF2 treatment were the lower levels of PAX6 expression and the upregulation of CDX2 observed with FGF2. PAX6 is frequently used as a marker of hESC neuralization, but it is more abundantly expressed in the anterior regions of the early neuroectoderm *in vivo* [32], whereas CDX2 is expressed in the developing posterior neuroectoderm as well as non-neural tissues [33]. Based on the analysis of several other markers of neural and non-neural fates and neural patterning, while we cannot exclude that CDX2 upregulation indicates mesoderm differentiation in SB + FGF2-treated cultures, we speculate that the differential regulation of PAX6 and CDX2 by FGF2 may not simply be due to inefficient neuralization, but may at least in part reflect the different AP positional identity of neuroectoderm induced by Noggin and FGF2. We found that FGF2-induced neuroectoderm was strongly posteriorized, displaying robust upregulation of hindbrain/spinal cord genes without significant activation of forebrain genes. While this posteriorizing effect was partially dependent on activation of Wnt/β-catenin signalling, FGF2 could also exert a direct posteriorizing influence on hESC-derived neuroectoderm, which was not prevented by extracellular Wnt antagonists. Preliminary analyses of the timing of FGF-mediated posteriorization showed that, whereas FGF2 started to promote posterior specification after the first 2 days of differentiation, treatment of Noggin-induced neuroectoderm with FGF2 after 6–8 days of differentiation still caused partial posteriorization (data not shown). These observations support studies in vertebrate model systems suggesting that FGF signalling can modulate AP patterning independently of an earlier function as a neural inducer [2,3]. These posteriorizing effects occurred at doses of FGF2 within the range (10–20 ng ml^−1^) routinely used for expansion of neural stem/progenitor cells. Thus, FGF2 does not simply act as a mitogen on neural progenitors, and alternative growth factors should be considered for expansion of progenitors with rostral identities, as confirmed by the observation that hESC-derived neural stem cells quickly lose forebrain gene expression when expanded in the presence of FGF2 and instead settle on hindbrain positional identities [54].

Assays initially performed in *Xenopus* ectodermal explants [44] and recently also validated in hESCs [16–18] have led to the idea that BMP antagonists induce neuroectoderm with forebrain identity, which can be posteriorized by caudalizing factors, such as Wnts, FGFs and RA. Supporting this notion, we show that differentiation of adherent, chemically defined hESC cultures in the presence of Noggin resulted in anterior neural specification, with little or no upregulation of hindbrain/spinal cord markers. We found, however, a mild degree of posteriorization in Noggin-induced neuroectoderm, as shown by upregulation of midbrain markers along with forebrain markers. Midbrain gene expression in Noggin-treated cultures involved activation of Wnt/β-catenin signalling and could be noticeably reduced (and forebrain gene expression enhanced) by β-catenin inhibitors, even in the presence of Wnt ligand inhibitors. This suggests that Wnt-independent β-catenin signalling may be active in our culture conditions and exert a mild posteriorizing influence even when Wnt activities are inhibited. While more work will be needed to elucidate the nature of the Wnt-independent pathways promoting β-catenin activation, small molecule β-catenin inhibitors are promising tools to protect hESC-derived forebrain from posteriorization in different culture environments.

FGF2 treatment in the presence of Wnt antagonists and β-catenin activation in the absence of exogenous FGF2 both caused mild posteriorization of hESC-induced neuroectoderm, whereas strong effects were determined by FGF activation in the absence of Wnt inhibitors. We found a slight increase in *LEF1* expression in cells treated with SB + FGF2 compared with cells treated with SB or SB + Noggin (data not shown), suggesting that, although FGF activation may promote Wnt signalling to a limited extent, these two pathways are more likely to interact in a cooperative fashion than in a simple linear sequence. Previous studies suggest the existence of such interactions [55], but certainly this is a topic deserving more thorough investigation.

While there is already extensive literature on neural differentiation of hESCs, generation of specific neural cell types from hESCs has mainly relied on application of pre-existing knowledge or on empirical approaches, with limited efforts made to unravel novel mechanisms of regional specification in the CNS. Owing to its complexity, forebrain regionalization remains the least understood aspect in neural patterning. A key event in this process is the segregation of the rostral forebrain into the presumptive telencephalon and the eye field [3]. While it is clear that both territories are specified in a region of low or absent Wnt activity [37,56,57], the signals responsible for partitioning this low-Wnt area into telencephalic- and retina-forming domains are still largely unknown.

Our results show that constitutive inhibition of Activin/Nodal and BMP signalling during hESC differentiation to neuroectoderm in adherent, defined conditions is compatible with the specification of telencephalic/diencephalic fates, but such inhibition clearly restrains upregulation of eye field genes. Temporally controlled treatments showed that Activin/Nodal inhibition during the first 4 days of differentiation impaired eye field gene expression. As SB treatment was largely dispensable for anterior neuroectoderm specification, Activin/Nodal inhibitors may be best avoided in protocols aimed at retinal cell generation in this system. Similar experiments for the BMP pathway showed a more complex situation. Upregulation of eye field genes was not hampered when Noggin treatments were limited to the first 6–8 days of differentiation, raising the possibility that a limited pulse of BMP inhibition may elicit neural induction without interfering with eye field specification. Neuroectoderm markers, however, were partially upregulated by Noggin treatments restricted to either the first or second week of differentiation, indicating that BMP inhibition is needed beyond the first 8 days for the stabilization of neural fates. A finer manipulation of both the timing and the levels of BMP inhibition, along with investigation of the molecular mechanisms of eye field gene regulation by BMP signalling, will be needed to achieve optimal experimental conditions for both neural induction and eye field specification.

In conclusion, we show that BMP, FGF and Wnt/β-catenin signalling pathways are crucial determinants of the specification of neuroectoderm with different rostrocaudal identities. Hence, we have validated, in defined cultures of human embryonic-like progenitors, the key mechanisms of neural patterning that are found in vertebrate model organisms such as *Xenopus*. Moreover, we provide new insights into the signals regulating further forebrain patterning and in particular how proper modulation of Activin/Nodal and BMP signalling may be critical for eye field specification.

## 6. Acknowledgements

We thank G. Augusti-Tocco, S. Casarosa and F. Cremisi for comments on this manuscript. We are grateful to members of our laboratories for support and advice. This work was supported by the Biotechnology and Biological Sciences Research Council (W.A.H., G.L.), the Wellcome Trust (W.A.H.), the Italian Ministry of Education, University and Research programme ‘Rientro dei Cervelli’ and a start-up grant from Istituto Pasteur-Fondazione Cenci Bolognetti (G.L.) and the Medical Research Council (R.A.P., L.V.).

## Supplementary Material

Supplementary material
