# Supplementary material for: Multiple roles of Activin/Nodal, bone morphogenetic protein, fibroblast growth factor and Wnt/β-catenin signalling in the anterior neural patterning of adherent human embryonic stem cell cultures
Source: Open Biol. 2013 Apr;3(4):120167. doi: 10.1098/rsob.120167 (PMC3718331; doi:10.1098/rsob.120167)
Supplement: Supplementary material [file rsob120167-s1.pdf]

SUPPLEMENTARY FIGURE LEGENDS

Figure S1. Expression of tissue-specific markers in adherent hESC cultures treated with SB, SB+Noggin or SB+FGF2.

(A,B) Real time PCR quantification of gene expression in hESCs cultured for 12-16 days with SB, SB+N or SB+F, as indicated. Compared to SB or SB+F treatments, SB+N-treated cells show reduced transcription of the non-neural markers *SOX7*, *CDX2*, *SOX17* and the neural crest marker *SOX10*. No major differences are detectable in the expression of the neuroectoderm markers *NCAM* and *SOX3*. Results are shown as the mean of the Log_{10}-transformed ratio between SB, SB+N or SB+F and A+F conditions in three biological replicates.

Figure S2. Expression of tissue-specific markers in adherent hESC cultures treated with SB, SB+Noggin or SB+FGF2.

(A,B) Real time PCR quantification of gene expression in hESCs cultured for 12-16 days with SB, SB+N or SB+F, as indicated. Compared to SB treatments, cells treated with SB+N or SB+F show reduced transcription of the trophoectoderm marker *HAND1*. No major differences are detectable in the expression of the mesoderm markers *LMO2*, *FLK1* and *T* and the trophoectoderm marker *EOMES*, while the expression of mesoderm marker *TBX6* is slightly higher in SB+F-treated cells . Results are shown as the mean of the Log_{10}-transformed ratio between SB, SB+N or SB+F and A+F conditions in three biological replicates.

Figure S3. Opposite effects of Wnt5a and Wnt3a on AP patterning of adherent hESCs differentiated to neuroectoderm with SB+FGF2.

RT-PCR analysis of gene expression in hESCs treated for 8-20 days with 20 SB+FGF2, with or without Wnt5a or Wnt3a. Wnt5a and Wnt3a counteract or accelerate, respectively, neuroectoderm posteriorization in SB+FGF2-treated cells.

Figure S4. Exogenous FGF2 promotes expression of posterior neural markers in adherent hESCs differentiated in the presence of Wnt antagonists.

RT-PCR analysis of gene expression in hESCs treated for 8-16 days with SB, Noggin, Fzd8 (SB+NOG+Δ8) and 12-120 ng/ml FGF2 (FGF 12-120). Expression of hindbrain/spinal cord markers becomes more noticeable at higher FGF doses.

Figure S5. Endogenous Hh signalling is active in adherent hESC cultures and promotes neuroectoderm ventralization

RT-PCR analysis of gene expression in hESCs treated for 12-16 days with SB+N+Δ8, with or without the Hh-pathway agonist Purmormaphine (PMP) or the Hh-pathway antagonist Cyclopamine, used at 2-4 μM (CPM2-4). Hh signalling is endogenously active in undifferentiated hESCs and during their differentiation with SB+N+Δ8, as shown by transcription of *GLI1*, a direct target gene of Hh signalling, in undifferentiated hESCs and, although more weakly, at 12-16 days of differentiation. When hESCs treated with SB+Noggin+Fzd8Δ were also exposed to PMP or CPM, upregulation of *NKX2.1* expression during differentiation was further enhanced or strongly repressed, respectively, while *VAX1* or *FOXG1* expression was similar in all conditions.

Figure S6. Effects of SB and Noggin treatments on the expression of tissue-specific and pluripotency markers in adherent hESC cultures.

(A-C) Real time PCR quantification of gene expression in hESCs treated for 12-16 days with Δ8, with or without SB and/or Noggin, as indicated. Noggin treatments cause reduced transcription of *SOX7*, *CDX2*, *SOX17* and *SOX10*. No major differences are detectable in the expression levels of the pluripotency markers *NANOG* and *OCT4* and neuroectoderm markers *NCAM* and *SOX3*, while *SOX2* expression is higher in Noggin-treated cells. Results are shown as the mean of the Log_{10}-transformed ratio between differentiated and undifferentiated hESCs in four biological replicates.

Figure S7. Temporal analysis of the effects of SB treatments on the expression of eye field genes in anterior neuroectoderm derived from adherent hESC cultures.

(A) Left, schematic representation of the timely controlled manipulation of Activin/Nodal signalling during anterior neuroectoderm differentiation of hESCs. Cells were treated treated for 12 days with Noggin and Δ8 (N+Δ8), with or without SB. In alternative, SB was either removed after 4 days, 6 days or 8 days and replaced with low doses of Activin (Ad4-8), or SB was added only after 4 days, 6 days or 8 days (SBd4-8). Right, real time PCR quantification of *LEFTY2* expression in these assays, showing that Activin treatments restore the levels of Activin/Nodal signalling observed in the absence of SB. Numbers above the bars correspond to the treatment conditions shown in the scheme. Results are shown as the mean of the Log_{10}-transformed ratio between differentiated and undifferentiated hESCs in four biological replicates.

(B,C) Real time PCR quantification of eye field gene expression in the same assays as in **(A)**, showing that SB treatments repress *RAX* and *LHX2* expression during the first

four days of differentiation, while *SIX6* and *VSX2* expression is affected over a longer time window.

Figure S8. Temporal analysis of the effects of Noggin treatments on the expression of eye field, telencephalic/diencephalic and neuroectoderm genes in differentiating adherent hESC cultures.

(A) Left, schematic representation of the timely controlled manipulation of BMP signalling during hECS differentiation. Cells were treated for 12 days with SB and Δ8 (SB+Δ8), with or without Noggin (N). Alternatively, Noggin was either removed after 4 days, 6 days or 8 days and replaced with low doses of BMP4 (Bd4-8), or Noggin was added only after 4 days, 6 days or 8 days (Nd4-8). Right, real time PCR quantification of *ID1* expression in these assays, showing that BMP4 treatment restore the levels of BMP signalling observed in the absence of Noggin. Numbers above the bars correspond to the treatment conditions shown in the scheme. Results are shown as the mean of the Log_{10}-transformed ratio between differentiated and undifferentiated hESCs in four biological replicates.

(B,C) Real time PCR quantification of eye field (*SIX6*, *VSX2*), telencephalic/diencephalic (*NKX2.1*, *VAX1*) or neuroectoderm (*SOX1*, *SOX2*) gene expression in the same assays as in **(A)**, showing that Noggin treatments repress eye field gene expression mainly after the first six-eight days of differentiation, while they promote *NKX2.1* expression mainly during the first four days and *VAX1* expression mainly after the first four days of differentiation. Results with *SOX1* and *SOX2* indicate that Noggin initiates neuroectoderm specification during the first four days of differentiation, but it is still required beyond day eight for the stabilization of neural fates.

Figure S1

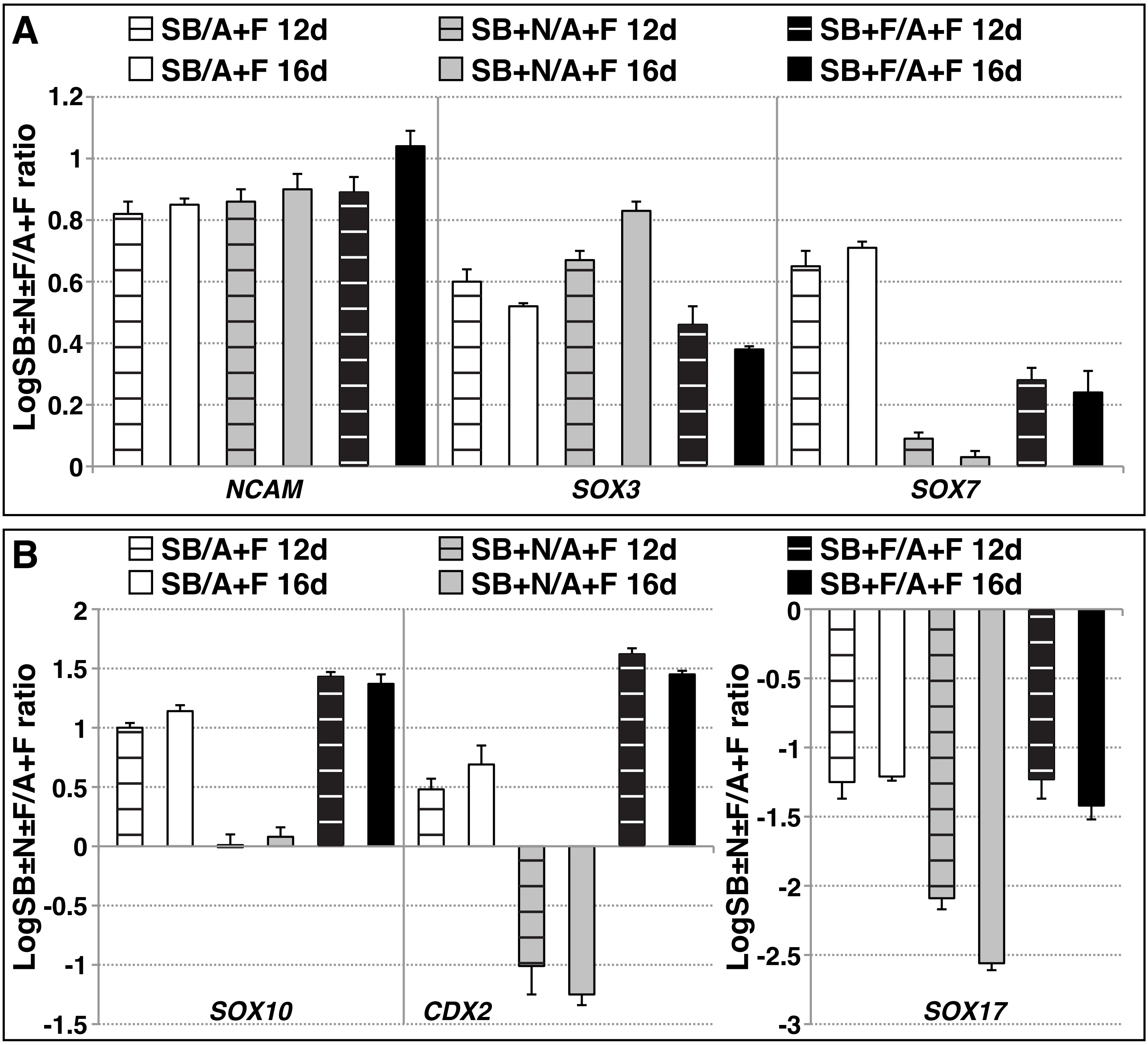

A
SB/A+F 12d
SB+N/A+F 12d
SB+F/A+F 12d
SB/A+F 16d
SB+N/A+F 16d
SB+F/A+F 16d
LogSB±N±F/A+F ratio
1.2
1
0.8
0.6
0.4
0.2
0
NCAM
SOX3
SOX7
B
SB/A+F 12d
SB+N/A+F 12d
SB+F/A+F 12d
SB/A+F 16d
SB+N/A+F 16d
SB+F/A+F 16d
LogSB±N±F/A+F ratio
2
1.5
1
0.5
0
-0.5
-1
-1.5
SOX10
CDX2
LogSB±N±F/A+F ratio
0
-0.5
-1
-1.5
-2
-2.5
-3
SOX17

Figure S2

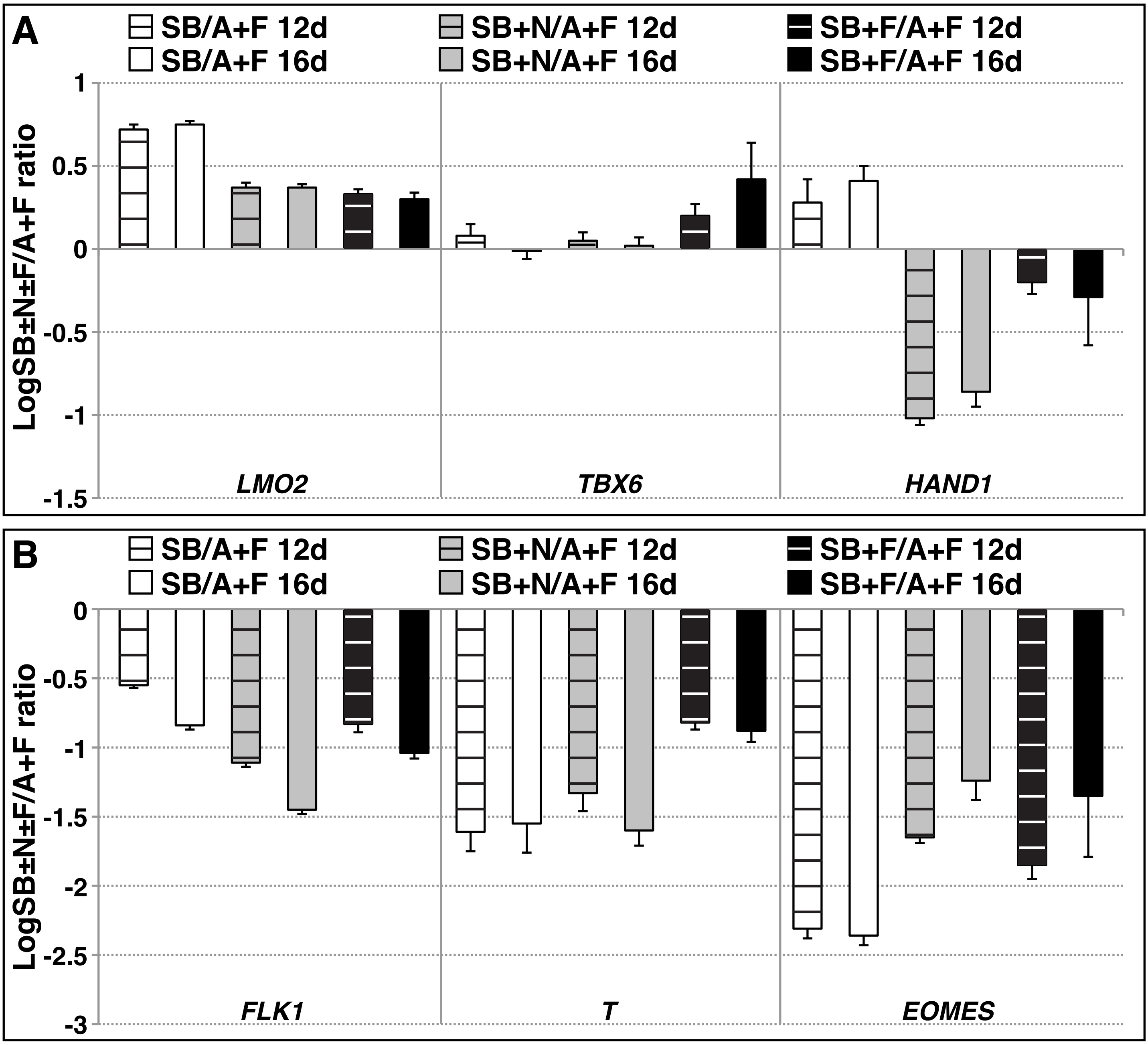

A
SB/A+F 12d
SB/A+F 16d
SB+N/A+F 12d
SB+N/A+F 16d
SB+F/A+F 12d
SB+F/A+F 16d
LogSB±N±F/A+F ratio
1
0.5
0
-0.5
-1
-1.5
LMO2
TBX6
HAND1
B
SB/A+F 12d
SB/A+F 16d
SB+N/A+F 12d
SB+N/A+F 16d
SB+F/A+F 12d
SB+F/A+F 16d
LogSB±N±F/A+F ratio
0
-0.5
-1
-1.5
-2
-2.5
-3
FLK1
T
EOMES

Figure S3

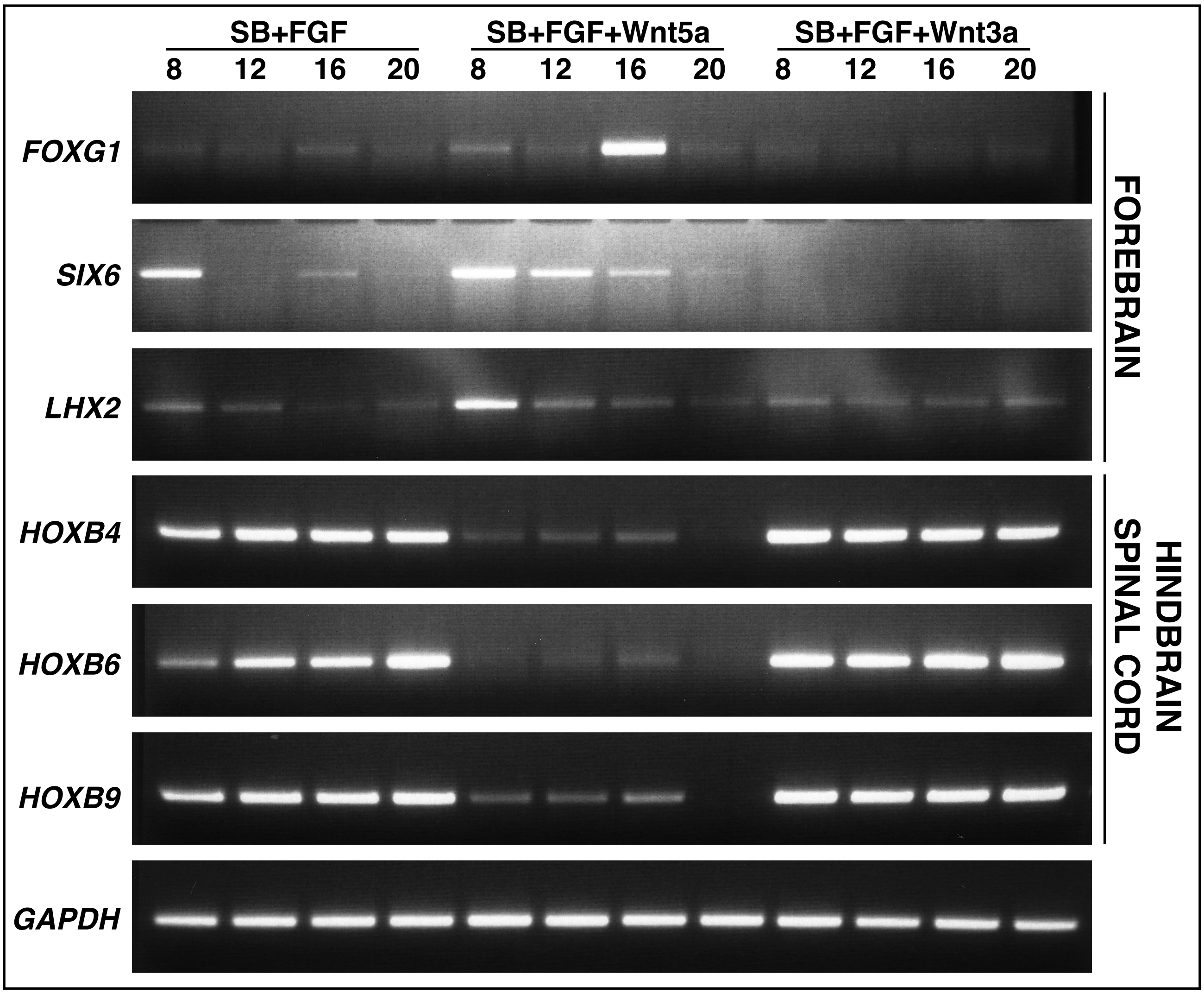
SB+FGF
SB+FGF+Wnt5a
SB+FGF+Wnt3a
8 12 16 20 8 12 16 20 8 12 16 20
FOXG1
SIX6
LHX2
HOXB4
HOXB6
HOXB9
GAPDH
FOREBRAIN
HINDBRAIN
SPINAL CORD

Figure S4

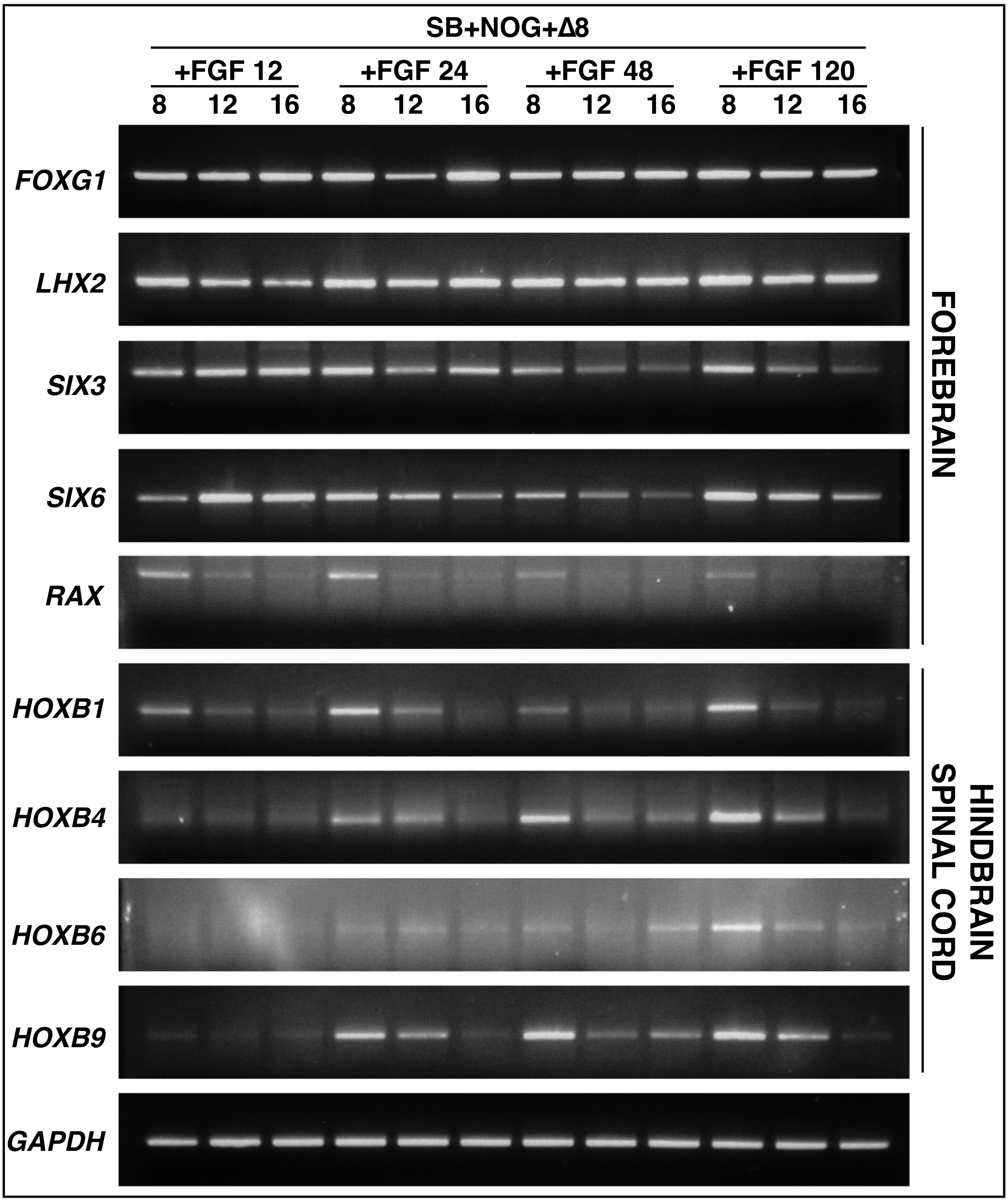

SB+NOG+Δ8
+FGF 12
+FGF 24
+FGF 48
+FGF 120
8 12 16 8 12 16 8 12 16 8 12 16
FOXG1
LHX2
SIX3
SIX6
RAX
HOXB1
HOXB4
HOXB6
HOXB9
GAPDH
FOREBRAIN
HINDBRAIN
SPINAL CORD

Figure S5

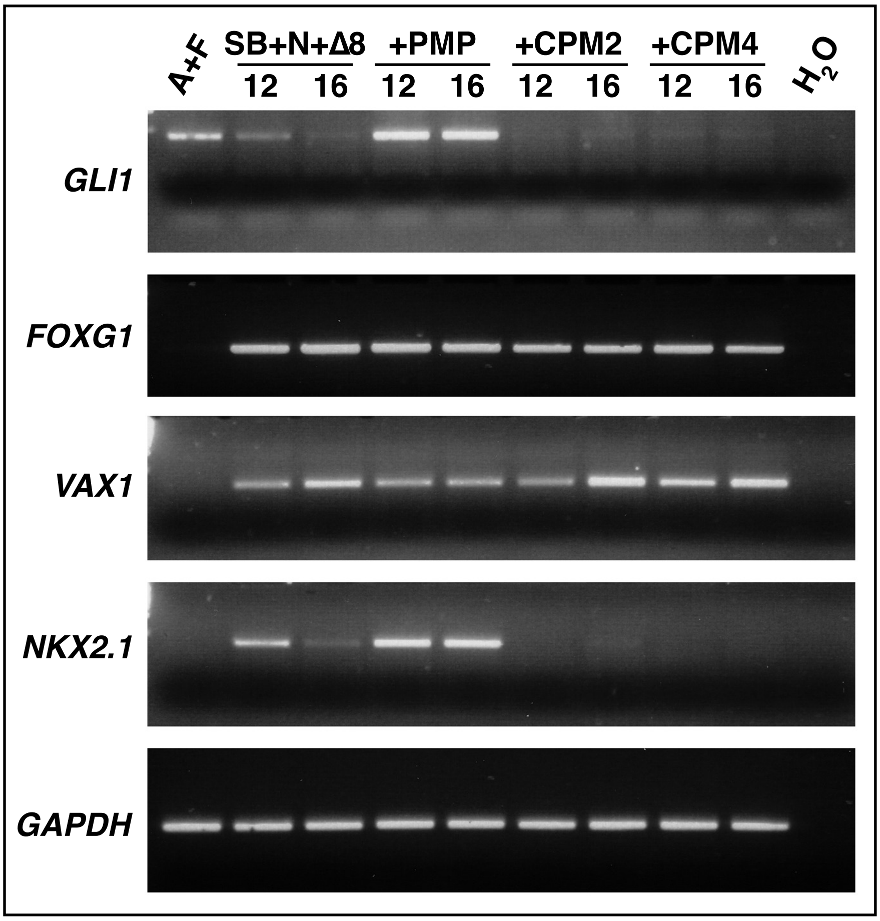
A+F
SB+N+Δ8
+PMP
+CPM2
+CPM4
12
16
12
16
12
16
12
16
H2O
GLI1
FOXG1
VAX1
NKX2.1
GAPDH

Figure S6

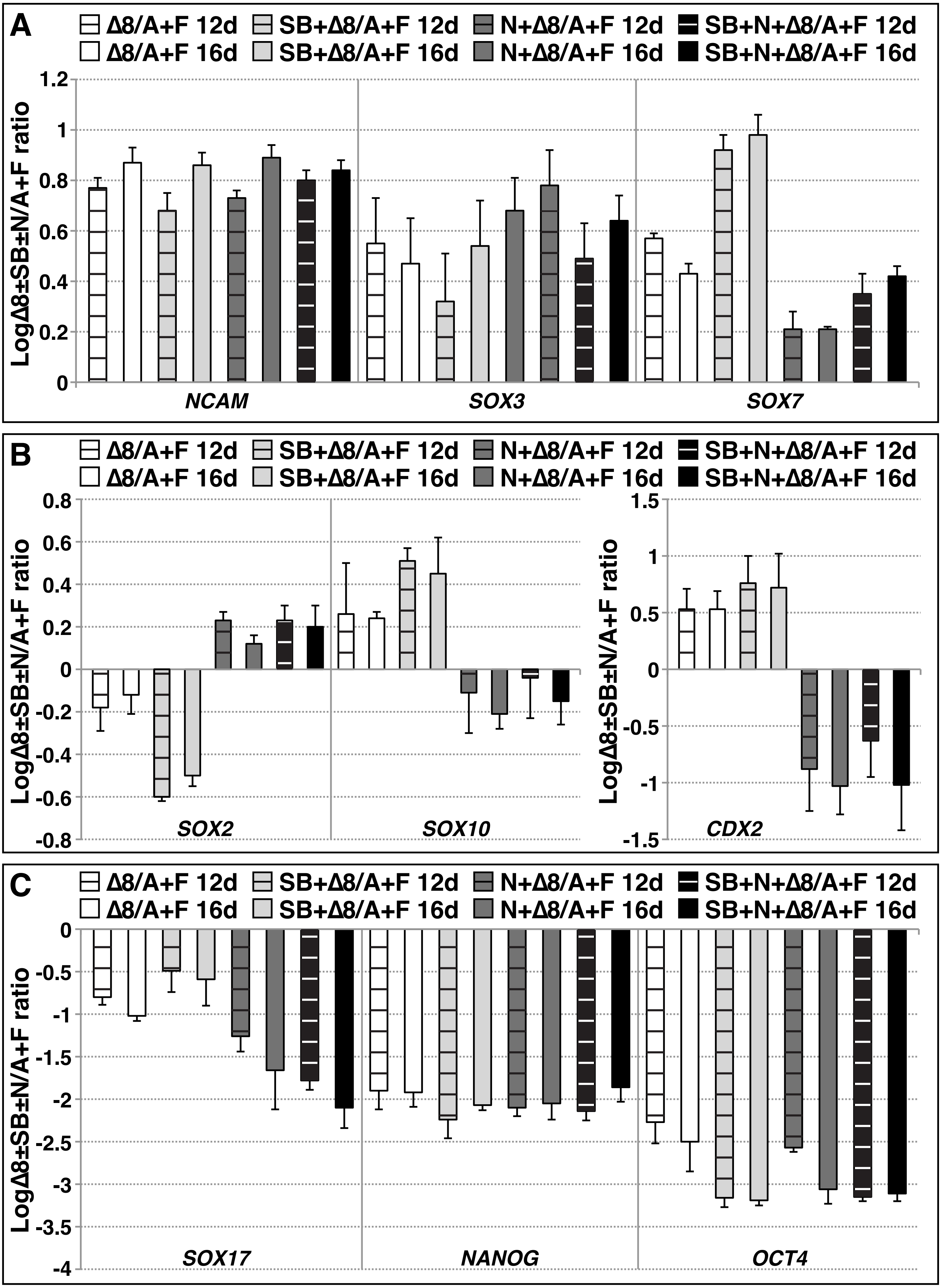
A
Δ8/A+F 12d
SB+Δ8/A+F 12d
N+Δ8/A+F 12d
SB+N+Δ8/A+F 12d
Δ8/A+F 16d
SB+Δ8/A+F 16d
N+Δ8/A+F 16d
SB+N+Δ8/A+F 16d
LogΔ8±SB±N/A+F ratio
NCAM
SOX3
SOX7
B
SOX2
SOX10
CDX2
C
SOX17
NANOG
OCT4

Figure S7

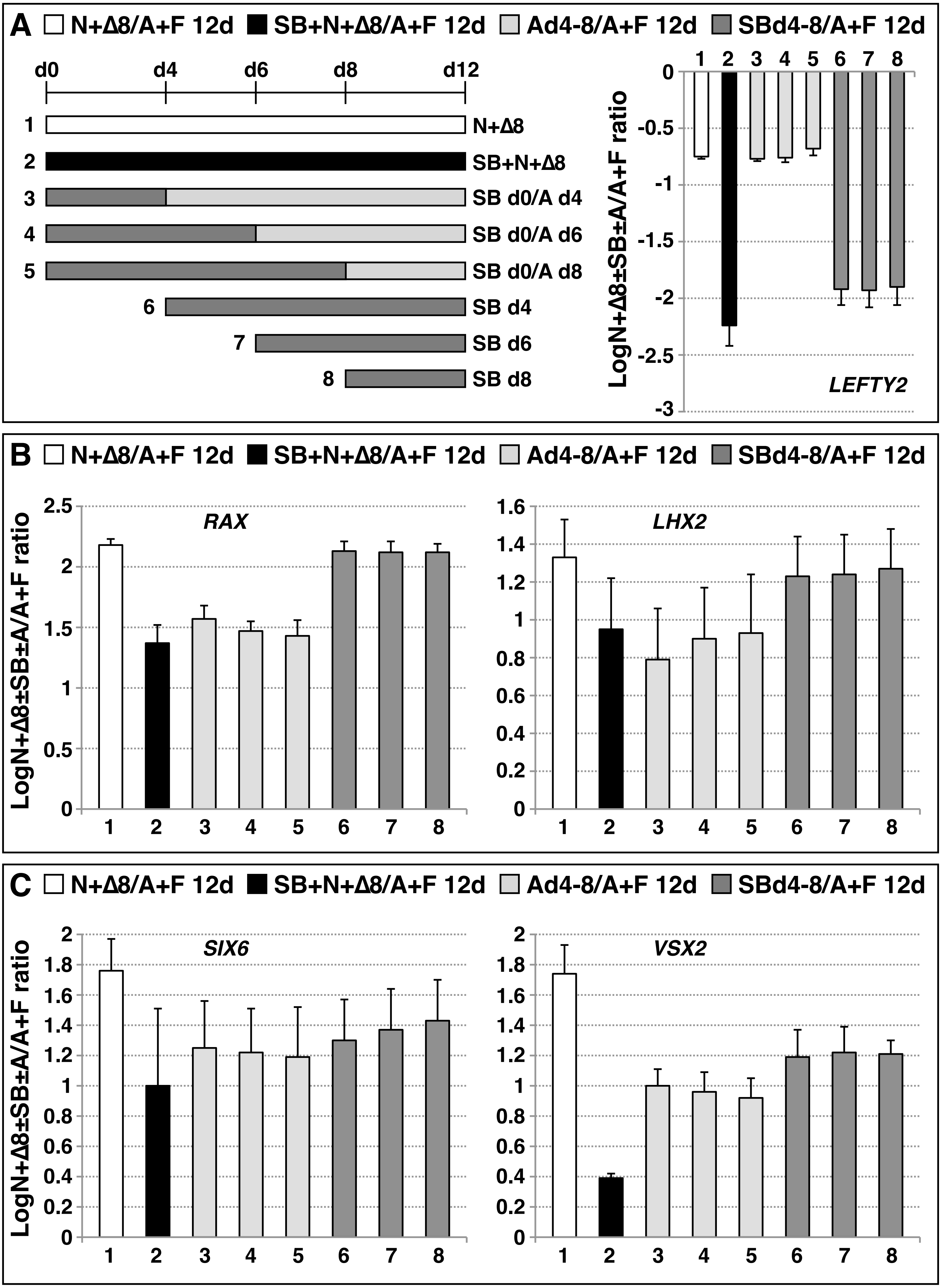
A
N+Δ8/A+F 12d
SB+N+Δ8/A+F 12d
Ad4-8/A+F 12d
SBd4-8/A+F 12d
d0
d4
d6
d8
d12
1 N+Δ8
2 SB+N+Δ8
3 SB d0/A d4
4 SB d0/A d6
5 SB d0/A d8
6 SB d4
7 SB d6
8 SB d8
LogN+Δ8±SB±A/A+F ratio
LEFTY2
0
-0.5
-1
-1.5
-2
-2.5
-3
1 2 3 4 5 6 7 8
B
N+Δ8/A+F 12d
SB+N+Δ8/A+F 12d
Ad4-8/A+F 12d
SBd4-8/A+F 12d
LogN+Δ8±SB±A/A+F ratio
RAX
2.5
2
1.5
1
0.5
0
LHX2
1.6
1.4
1.2
1
0.8
0.6
0.4
0.2
0
1 2 3 4 5 6 7 8
C
N+Δ8/A+F 12d
SB+N+Δ8/A+F 12d
Ad4-8/A+F 12d
SBd4-8/A+F 12d
LogN+Δ8±SB±A/A+F ratio
SIX6
VSX2
2
1.8
1.6
1.4
1.2
1
0.8
0.6
0.4
0.2
0
1 2 3 4 5 6 7 8

Figure S8

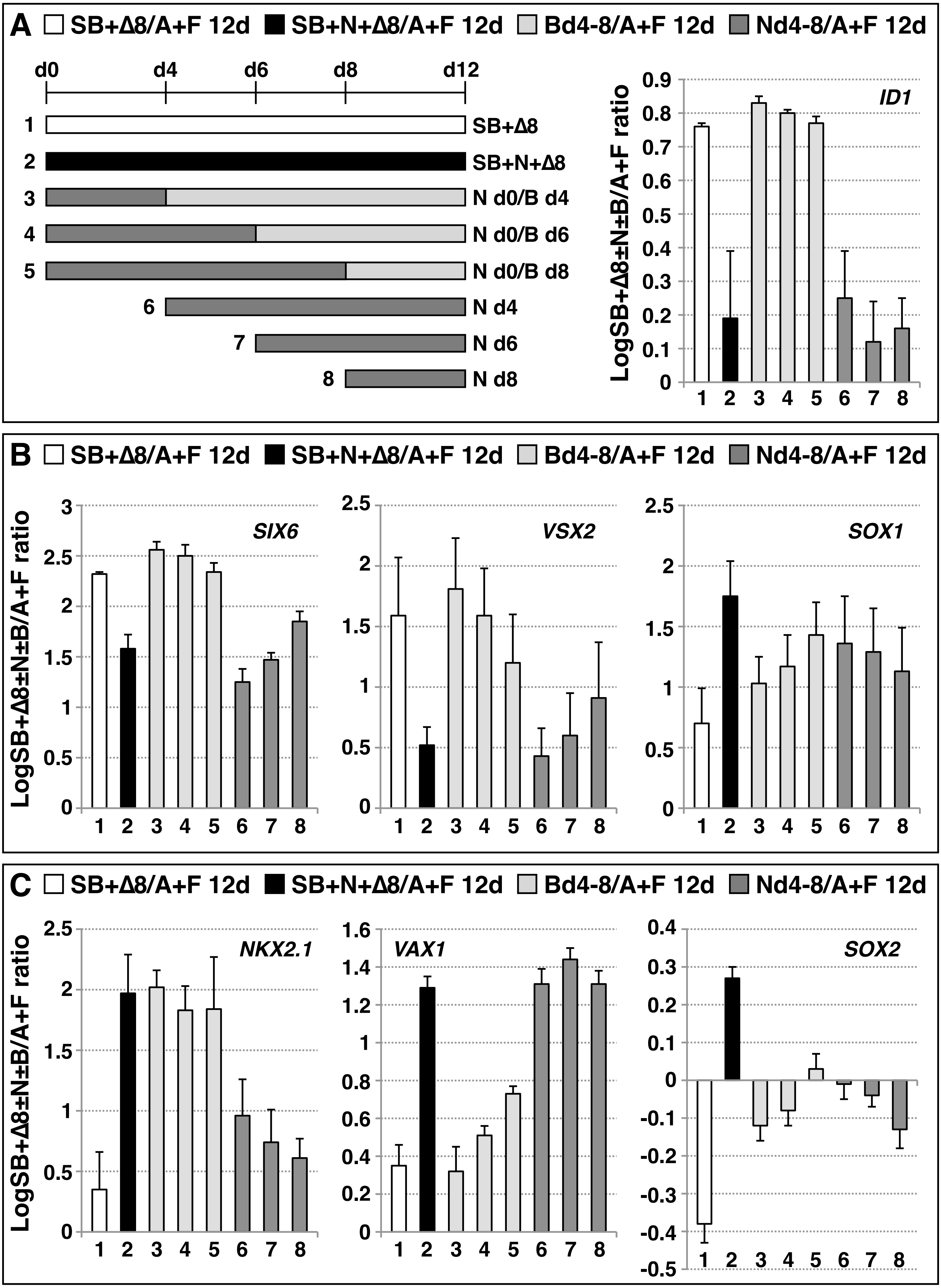
A
SB+Δ8/A+F 12d
SB+N+Δ8/A+F 12d
Bd4-8/A+F 12d
Nd4-8/A+F 12d
d0
d4
d6
d8
d12
SB+Δ8
SB+N+Δ8
N d0/B d4
N d0/B d6
N d0/B d8
N d4
N d6
N d8
ID1
LogSB+Δ8±N±B/A+F ratio
B
SIX6
VSX2
SOX1
C
NKX2.1
VAX1
SOX2

**Molecule**	**Source**	**Concentration used**
Activin	Produced in-house	10-20 ng/ml (feeder-free hESC culture)<br>5 ng/ml (timing assays)
FGF2	Produced in-house	12 ng/ml (feeder-free hESC culture)<br>12-120 ng/ml (neuroectoderm differentiation
SB431542	Tocris (Cat.No. 1614)	10-20 µM
Noggin	R&D Systems (Cat.No. 3344-NG)	250-375 ng/ml
BMP4	Produced in-house	5 ng/ml
Frizzled8Δ	R&D Systems (Cat.No. 112-FZ)	200-1250 ng/ml
Dkk1	R&D Systems (Cat.No. 1096-DK)	100 ng/ml
XAV939	Tocris (Cat.No. 3748)	1-2 µM
Wnt3a	R&D Systems (Cat.No. 1324-WN)	20 ng/ml
Wnt5a	R&D Systems (Cat.No. 645-WN)	100 ng/ml
Cyclopamine	TRC (Cat. No. C988400)	2-4 µM
Purmorphamine	Merck (cat. No. 540220)	1 µM
